# Inhibition of NLRP3 inflammasome activation by caffeine might be a potential mechanism to reduce the risk of squamous cell carcinoma of the oral cavity and oropharynx with coffee drinking

**DOI:** 10.3389/froh.2022.1017543

**Published:** 2022-09-22

**Authors:** Frank S. Fan

**Affiliations:** Section of Hematology and Oncology, Department of Medicine, Ministry of Health and Welfare Taitung Hospital, Taitung City, Taiwan

**Keywords:** caffeine, coffee, NLRP3 inflammasome, oral cavity, oropharynx, squamous cell carcinoma

## Introduction

According to the GLOBOCAN 2020 assessment of cancer statistics generated by the International Agency for Research on Cancer, cancer of the lip and oral cavity is the 18th most common cancer worldwide, with 377,713 new cases and 177,757 new deaths worldwide in 2020, while cancer of the oropharynx is the 26th most common cancer worldwide, with 98,412 new cases and 48,143 new deaths in 2020 (1). Most of the two cancers are squamous cell carcinomas (SCC) in pathology classification, and Asia has the highest incidence and mortality rates for both cancers (2, 3). Notably, oral SCC is the most frequently diagnosed cancer in South Asian countries, including India, Pakistan, Sri Lanka, and Bangladesh, and prevention strategies are urgently required (4). The consumption of tobacco, alcohol, and betel quid has been reported to be a major risk factor for oral SCC, and the reduction of their use has been the main focus of cancer control in relevant areas (5). Meanwhile, interestingly, in epidemiological studies, a common drink, coffee, has long been found to have preventive potential against both the oral cavity and oropharyngeal SCC. The impact of coffee on the risk of various cancers has been intensively investigated in many case-control and cohort studies. Meta-analysis of these observational studies led to the conclusion that coffee intake is inversely related to the occurrence of oral cavity and oropharyngeal cancers (6, 7). However, the protective influence of coffee on cancer of the oral cavity and oropharynx appeared to exist only in caffeinated coffee (8). The molecular mechanism underlying the chemopreventive effects of coffee on cancer remains unclear and needs further investigation (9). Herein, a hypothesis is proposed to explain the beneficial effects of coffee on oral and oropharyngeal cancer based on a thorough literature review of recent progress in the field of inflammasome research.

## NLRP3 inflammasome and oral cavity/oropharynx cancer

Inflammasomes are a group of cytoplasmic multiprotein complexes activated by harmful stimuli called damage-associated molecular patterns or pathogen-associated molecular patterns that elicit caspase-1, leading to downstream inflammatory pathways. An inflammasome is defined by its pattern-recognition receptors, such as the nucleotide-binding oligomerisation domain and leucine-rich repeat (LRR)-containing protein family member, NLRP3 (10). Inflammasome activation results in the secretion of the inflammatory mediators interleukin (IL)-1β and IL-18, apoptosis, and pyroptosis. Proper activation of inflammasomes helps the host deal with infectious pathogens or tissue damage. In contrast, anomalous inflammasome activation can induce a deviating tissue response that may lead to autoimmune disorders, neurodegenerative diseases, cardiometabolic abnormalities, and cancer (11).

The roles of NLRP3 inflammasome activation in carcinogenesis and tumour growth promotion in oral SCC have been well demonstrated in three modern rigorous studies carried out in one immortalised human oral epithelial cell line and several oral SCC cell lines. The first showed that the components of the NLRP3 inflammasome were highly expressed in these cell lines and correlated tightly with the characteristic markers of cancer stem cells of head and neck SCC. Sphere-forming and colony formation capacities of the cells were promoted upon activation of the NLRP3 inflammasome, but were reduced along with NLRP3 inflammasome blockade by an NLRP3 inhibitor. Furthermore, NLRP3 inflammasome blockade can delay tumour-burdened speed in transgenic head and neck SCC mice (12).

The second study also revealed high expression of NLRP3 in cell lines and paraffin-embedded human oral SCC tissues, with a good correlation between NLRP3 expression levels, tumour size, and lymph node metastatic status. NLRP3 knockdown inhibits the proliferation, migration, and invasion of oral SCC cells and reduces tumour growth *in vivo* (13). The third study found that IL-6 was upregulated in oral SCC tissues with a high expression level closely related to advanced tumour size, stage, and poorer survival, while IL-6 could stimulate proliferation and NLRP3 inflammasome activation through the JAK2/STAT3/Sox4 pathway in oral SCC cells, implying that the NLRP3 inflammasome is a therapeutic target in oral SCC (14). Furthermore, a fourth study revealed that activation of the NLRP3 inflammasome was increased in tumour specimens from patients who had received fluorouracil chemotherapy and oral SCC cells after fluorouracil treatment. Silencing of the NLRP3 inflammasome dramatically inhibits proliferation and enhances fluorouracil-induced apoptosis in oral SCC cells (15).

## Caffeine and NLRP3 inflammasome

Caffeine has been found to inhibit the NLRP3 inflammasome in various laboratory cell and animal models. A study in lipopolysaccharide-induced macrophages from a human monocyte leukaemia cell line revealed that caffeine inhibited NLRP3 inflammasome activation and thus decreased the secretion of IL-1β and IL-18 by suppressing mitogen-activated protein kinase/NF-κB signalling and adenosine A2a receptor (A2aR)-associated reactive oxygen species production (16). Another investigation in neonatal mice revealed that caffeine can reduce apoptosis of type 2 alveolar epithelial cells, preventing hypoxia-induced lung injury through inhibition of the NLRP3 inflammasome and the NF-κB pathway (17). Furthermore, caffeine appears to protect the central nervous system from various insults through similar mechanisms. Sepsis-associated encephalopathy can be protected by caffeine by inhibiting the uncoupling protein 2-mediated NLRP3 inflammasome pathway, leading to decreased neuronal apoptosis and mitochondrial dysfunction in astrocytes (18). The attenuation of microglia-mediated neuroinflammation in experimental autoimmune encephalomyelitis can be achieved by caffeine, which promotes autophagy and inhibits the activation of the NLRP3 inflammasome in microglia (19). Caffeine also improves microglial polarisation and long-term cognitive function in neonatal rats with hypoxic-ischaemic white matter damage through A2aR-mediated inhibition of NLRP3 inflammasome activation (20). Therefore, it is assumed that inhibiting inflammasome activation by caffeine with the consequent anti-inflammatory and anti-pyroptosis effects could provide beneficial effects in combating ageing (21) and severe acute respiratory syndrome coronavirus 2 infection (22).

## Hypothesis and discussion

Integrating all the epidemiological data and experimental results listed above, it is logical to assume that coffee reduces the risk of the oral cavity and oropharynx SCC due to the ability of caffeine to inhibit NLRP3 inflammasome activation, thus suppressing carcinogenesis and eliminating stem cells. Additionally, inactivation of the NLRP3 inflammasome can interrupt the proliferation, migration, and invasion of oral SCC cells. Although this speculation provides a reasonable explanation for the beneficial impact of coffee on the oral cavity and oropharyngeal SCC, further carefully designed studies are needed to test its reality.

However, the incidence of two other SCC malignancies arising from the upper digestive and respiratory tracts, oesophageal and laryngeal cancer, did not have a similar reverse correlation with coffee consumption despite the same risk factors for the disease, such as smoking and alcohol consumption (23, 24). It is currently unknown whether NLRP3 inflammasome activation plays a role in carcinogenesis at these two sites. The molecular carcinogenic mechanisms may not be identical for SCC from different anatomic sites.

Human papillomavirus (HPV) infection has been found to be a significant risk factor for oral cavity SCC in a systematic meta-analysis of randomised control trials, cross-sectional and cohort studies (25). Although the clinical characteristics and prognosis of HPV-positive and HPV-negative oropharyngeal cancer are quite different (26), the expression levels of NLRP3 did not show differences in human oropharyngeal SCC specimens, regardless of whether they contained HPV DNA (27). Activation of the NLRP3 inflammasome has been proposed as a key step in tumourigenesis induced by DNA oncogenic viruses such as HPV (28). Consequently, coffee drinking should bring beneficial cancer chemopreventive effects to people, regardless of their HPV infection status.

Finally, the direct effect of caffeine on cancer cell growth was examined in two cell lines established from tongue SCC and oesophageal SCC. Caffeine had remarkable inhibitory effects on both cell lines at experimental concentrations (29). These exciting results remind us of the possibility of using caffeine as an adjunctive regimen along with the main treatment in cancer therapy, especially for the oral cavity and oropharyngeal SCC. It is hoped that, with meticulous clinical trials, caffeine will be proven to be effective in cancer prevention and treatment in the near future.

## Conclusions

Epidemiological studies have found that the intake amount of caffeinated but not decaffeinated coffee has an inverse relationship with the incidence of the oral cavity and oropharyngeal SCC. NLRP3 inflammasome activation plays an important role in carcinogenesis and tumour progression in these two cancer types. Several experimental studies have shown that caffeine can inhibit NLRP3 inflammasome activation. Although current speculation is limited by the lack of experiments demonstrating the straightforward suppressive effects of caffeine on the NLRP3 inflammasome in cancer cells derived from oral cavity and oropharynx SCC and the subsequent inhibition of oral cancer growth, it is still confident that the cancer prevention effect of coffee on the oral cavity and oropharynx SCC stems from the inhibition of NLRP3 inflammasome activation by caffeine. There appears to be a great prospect that more laboratory work and clinical trials in this regard will eventually make people acknowledge the expedient efficacy of caffeine in preventing and treating oral cancer.
